# Novel Cu(I)-5-nitropyridine-2-thiol Cluster with NIR Emission: Structural and Photophysical Characterization

**DOI:** 10.1021/acs.jpcc.2c01842

**Published:** 2022-06-08

**Authors:** Khaled Hassanein, Chiara Cappuccino, Marianna Marchini, Elisa Bandini, Meganne Christian, Vittorio Morandi, Filippo Monti, Lucia Maini, Barbara Ventura

**Affiliations:** † Istituto per la Sintesi Organica e la Fotoreattività (ISOF), Consiglio Nazionale delle Ricerche (CNR), Via P. Gobetti 101, Bologna 40129, Italy; ‡ Dipartimento di Chimica “G. Ciamician”, Università di Bologna, Via F. Selmi 2, Bologna 40126, Italy; § Istituto per la Microelettronica e Microsistemi (IMM) Sede di Bologna, Consiglio Nazionale delle Ricerche (CNR), Via P. Gobetti 101, Bologna 40129, Italy

## Abstract

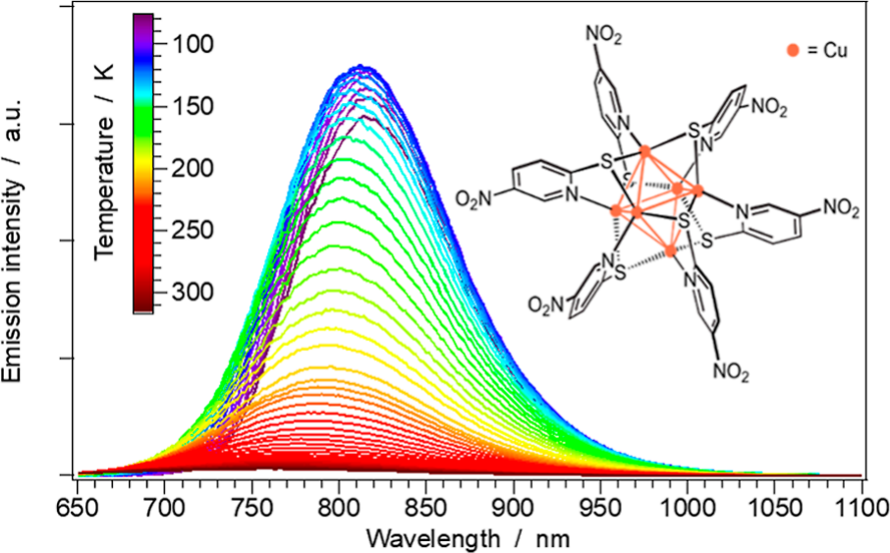

A novel Cu(I) cluster compound has been synthesized by reacting CuI with the 2,2′-dithiobis(5-nitropyridine) ligand under solvothermal conditions. During the reaction, the original ligand breaks into the 5-nitropyridine-2-thiolate moiety, which acts as the coordinating ligand with both N- and S-sites, leading to a distorted octahedral Cu_6_S_6_ cluster. The structure has been determined by single-crystal X-ray diffraction and FT-IR analysis, and the photophysical properties have been determined in the solid state by means of steady-state and time-resolved optical techniques. The cluster presents a near-infrared emission showing an unusual temperature dependence: when passing from 77 to 298 K, a blue-shift of the emission band is observed, associated with a decrease in its intensity. Time-dependent-density functional theory calculations suggest that the observed behavior can be ascribed to a complex interplay of excited states, basically in the triplet manifold.

## Introduction

Solid materials and coordination compounds emitting in the near-infrared (NIR) have emerged as promising and challenging materials with potential applications in optoelectronics, sensors, and telecommunications.^1−3^ Cu(I) compounds are a special class of materials for their remarkable photophysical and electronic properties.^4−10^ In particular, polynuclear Cu(I) clusters are an intriguing family of materials that are being investigated for promising applications in optoelectronics and luminescence signaling.^11−18^ Among them, hexacopper(I) clusters formulated as [Cu_6_S_6_], constitute a rarely explored family of polynuclear complexes, with high potential in the design of NIR luminescent materials.^19−23^

The challenging formation of Cu_6_S_6_ clusters can be afforded by treating the Cu(I) cation with ligands bearing −SH and −N moieties, such as those present in the mercaptonicotinic acid.^24^ In general, organosulfur ligands are frequently applied as bridging ligands for adjacent transition-metal sites in the design of luminescent materials.^25,26^ The incorporation of a thiolate as a bridge in transition-metal complexes, in fact, is highly promising in terms of luminescence output since it allows a suitable match of orbital energies between the ligand and the metal, leading to a large electronic delocalization.^27^

Moreover, ligands with a disulfide group have attracted a considerable interest in coordination chemistry, due to the facile *in situ* cleavage of the S–S bond under solvothermal conditions, which results in the preparation of structures showing a large variety of crystal arrangements and properties.^25,28^

In this work, the organic ligand 2,2′-dithiobis(5-nitropyridine) was chosen, owing to its rigid structure, multiple coordinated sites, and excellent coordination ability. The nitrogen atom can function as a bridging linker to construct high-dimensional structures. In addition, the sulfur and nitrogen atoms can synergistically chelate more than one metal ion to build multinuclear clusters. To the best of our knowledge, there are only two reports on complexes where the 2,2′-dithiobis(5-nitropyridine) has been used as a ligand, in one case bridging Ag(I) atoms in one-dimensional extended polymeric chains^29^ and in the other case acting as a single ligand coordinated to Ru(II) in its reduced form nitropyridylsulfide.^30^

We report here on the preparation and structural and photophysical characterization of cluster **1**, obtained by the reaction of CuI with 2,2′-dithiobis(5-nitropyridine) under solvothermal conditions. Combined luminescence studies at variable temperatures and comprehensive time-dependent-density functional theory (TD-DFT) calculations were employed to unravel the interplay of emissive triplet excited states that gives origin to the observed NIR emission.

## Experimental Methods

### Synthesis of [Cu(C_5_H_3_N_2_O_2_S)]_*n*_ (1)

A mixture of CuI (0.03 g, 0.16 mmol) and 2,2′-dithiobis(5-nitropyridine) (0.025 g, 0.08 mmol) was dissolved in 8 mL of DMF. Then, trimethylamine was added drop-by-drop over the orange suspension until it converted completely into a deep red suspension. This suspension was sealed in a 45 mL Teflon-lined steel autoclave, heated at 120 °C for 40 h and finally cooled to 20 °C in 24 h. Red crystals of **1** (0.012 g, 34% yield based on Cu) were filtered from the yellow solution and washed with H_2_O, MeCN, and diethyl ether and finally dried under vacuum. IR selected data (KBr, cm^–1^): 1589, 1556, 1495, 1443, 1331, 1267, 1138, 1092, 1078, 860, 835, 746, 550, 526, 476, 424.

Nanocrystals of **1** (**1n**) have been prepared using a fast precipitation method using water as the precipitating agent. 2,2′-dithiobis(5-nitropyridine) (0.025 g, 0.08 mmol) has been dissolved in 10 mL of MeOH, then triethylamine was added dropwise until the ligand was dissolved. CuI (0.03 g, 0.16 mmol) in 10 mL of CH_3_CN was added over this solution to give an orange/yellow solution. 100 mL of H_2_O has been added in one-pot step, giving rise immediately to a red solid formation, **1n** (0.01 g, 30% based on Cu).

### Absorption and Emission Spectroscopies

Room-temperature absorption and emission spectra were recorded with a Perkin-Elmer Lambda 950 UV/vis/NIR spectrophotometer and with an UV/vis Edinburgh FLS920 fluorimeter, equipped with a Peltier-cooled Hamamatsu R928 PMT (280–850 nm), respectively. Absorption and emission determinations were performed on powder samples placed between two quartz slides. Reflectance spectra were acquired with the spectrophotometer described above, equipped with a 100 mm integrating sphere. They have been converted into absorption spectra by using the Kubelka–Munk function.^31^ Emission spectra were collected in the front-face mode both with the UV/vis Edinburgh FLS920 fluorimeter and with the UV/vis/NIR FLS920 fluorimeter (Edinburgh) equipped with a Hamamatsu R5509-72 InP/InGaAs photomultiplier tube supercooled at 193 K in a liquid nitrogen cooled housing and a TM300 emission monochromator with a NIR grating blazed at 1000 nm (sensitivity range: 300–1700 nm). In both cases, the spectra have been corrected for the wavelength-dependent phototube response. For 77 K analysis, the samples were placed inside quartz capillary tubes and immersed in liquid nitrogen in a homemade quartz Dewar. For temperature-dependent measurements, the sample was mechanically spread on a quartz slide and placed inside an Oxford Optistat DN variable-temperature liquid-nitrogen cryostat (operating range: 77–500 K) equipped with an ITC5035 temperature controller and interfaced with the Edinburgh FLS920 fluorimeter. Excited-state lifetimes were determined with an IBH 5000F time-correlated single-photon-counting apparatus (pulsed NanoLED excitation source at 465 nm) or by using the Edinburgh FLS920 spectrometer equipped with a pulsed laser diode at 407 nm. The analysis of the luminescence decay profiles versus time was performed with a homemade program based on IgorPro software (WaveMetrics, Inc.). Absolute emission quantum yields were determined according to the method reported by Ishida *et al.*,^32^ by using a 4 in. Labsphere integrating sphere; the limit of detection of the system is 0.020. Estimated errors are 10% on exponential lifetimes, 20% on quantum yields, 20% on molar absorption coefficients, and 3 nm on emission and absorption peaks.

### Single-Crystal Diffraction

Single-crystal data for compound **1** were collected on an Oxford Xcalibur S equipped with Mo-Kα radiation (λ 0.71073 Å) and a graphite monochromator. The measurements of the crystal were performed at room temperature (298 K).

SHELXS was used for structure solution and SHELXL for the refinement based on F^2^ (Sheldrick, G. M. SHELXT—integrated space-group and crystal-structure determination).^33^ The non-hydrogen atoms were refined anisotropically, and the hydrogen atoms were added at calculated positions. All crystallographic data and further details on the data collection and structure determination are reported in Supporting Information (Table S1).

The experimental data were deposited within the Cambridge Crystallographic Data Centre (CCDC 2158477).

### SEM Analysis

Scanning electron microscopy (SEM) analyses were performed with a ZEISS LEO 1530 instrument equipped with a Schottky emitter, operated at an acceleration voltage of 5 keV, and In-lens detectors for secondary electron (SE) imaging.

### Powder X-ray Diffraction

X-ray powder diffraction (XRPD) analyses were performed on a PANalytical X’Pert Pro automated diffractometer with an X’Celerator detector in Bragg–Brentano geometry, using Cu Kα radiation (λ = 1.5418 Å), Soller slit of 0.04 rad, divergence slit of 1/4°, antiscatter slit of 1/2°, 40 mA, and 40 kV. Variable temperature XRPD were collected with the same instrument equipped with a non-ambient chamber Anton Paar TTK 450. TOPAS V7 was used for the Pawley refinement and determination of the average crystal domain size.^34,35^

### Computational Details

DFT calculations were carried out using the B.01 revision of the Gaussian 16 program package^36^ in combination with the M06 global-hybrid meta-GGA exchange–correlation functional.^37,38^ The fully relativistic Stuttgart/Cologne energy-consistent pseudopotential with multi-electron fit was used to replace the first 10 inner-core electrons of the copper metal centers (*i.e.*, ECP10MDF) and was combined with the associated triple-ζ basis set (*i.e.*, cc-pVTZ-PP basis).^39^ For sulfur atoms, the triple-ζ Pople 6-311G(2d) basis was selected;^40^ on the other hand, the smaller 6-31G(d) basis was adopted for all the other lighter atoms.^41^ Compound **1** was fully optimized using a time-independent DFT approach in its singlet ground state in vacuum. Frequency calculations were always used to confirm that the stationary point found by geometry optimization was actually a minimum on the corresponding potential-energy surface (no imaginary frequencies). TD-DFT calculations, carried out at the same level of theory used for geometry optimization, within the linear-response formalism, were used to calculate the first 64 singlets and 32 triplet excitations, and their nature was assessed by computing the density differences between the excited state of interest and the ground state. For the latter procedure, the excited-state densities were obtained by adding to the converged DFT wavefunction the necessary Z-vector contribution derived from a coupled-perturbed Kohn–Sham calculation to produce the relaxed density for the state and the excited state of interest.^42,43^

All the pictures showing geometries, molecular orbitals, and density differences were created using GaussView 6.^44^ The structural overlaps between different structures were obtained, thanks to the VMD program,^45^ by minimizing the root-mean-square deviation (RMSD) of all or selected atomic positions.

## Results and Discussion

### Synthesis and Structural Characterization

**Scheme 1:**
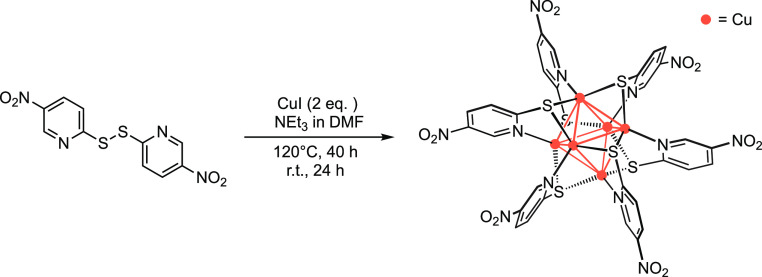
Chemical Sketch of the Synthesis of Complex **1** under Solvothermal Condition

The direct reaction of CuI with 2,2′-dithiobis(5-nitropyridine) ligand under solvothermal conditions gives rise to the formation of the novel thiolated Cu(I) cluster (**1**) where the S–S bond of the initial ligand has broken, most likely through an *in situ* reductive cleavage,^28,46^ to yield the new anionic 5-nitropyridine-2-thiolate ligand (Scheme 1).

**Figure 1:**
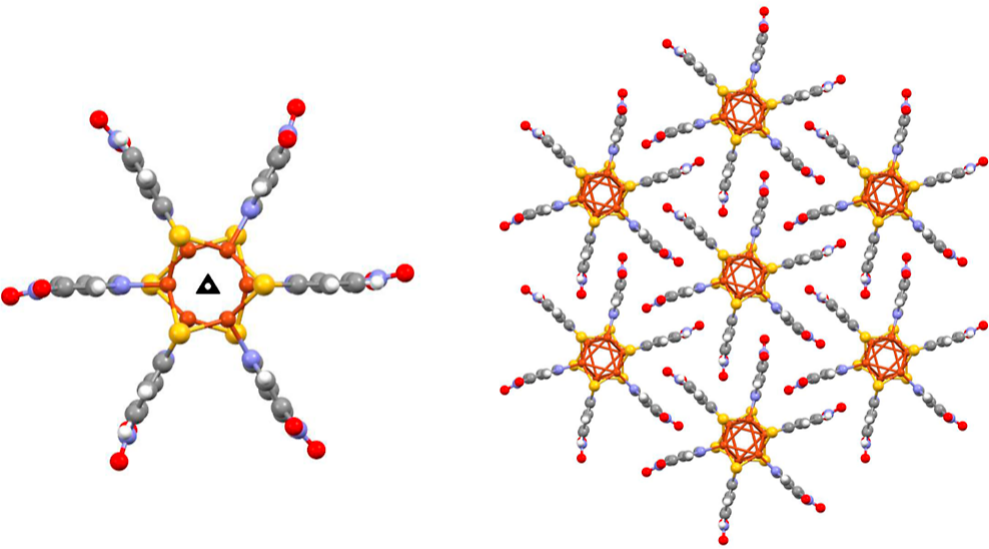
Cluster of Cu(I) viewed along the three-fold inversion axes in **1** (left). Packing structures of **1** viewed along the *c*-axes (right).

Single-crystal analysis revealed that compound **1** crystallizes in the trigonal *R*3 space group (Table S1). The asymmetric unit consists of one copper atom and one ligand, and the three-fold inversion axes generate the cluster, formed by six Cu(I) centers bridged by six 5-nitropyridine-2-thiol anions and figures as a distorted octahedron, as shown in Figure 1.

In the cluster, each Cu(I) is connected to two sulfur atoms and one nitrogen atom from three different 5-nitropyridine-2-thiol molecules, with Cu–S distances of 2.249–2.250 Å and Cu–N distance of 2.053 Å.

**Figure 2:**
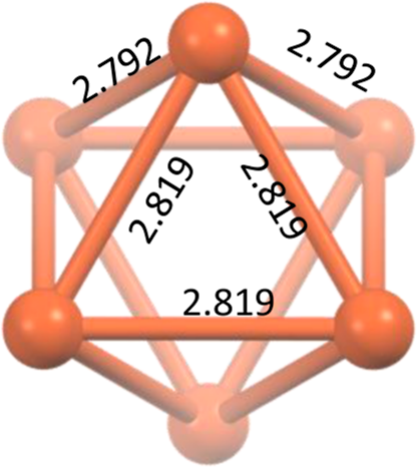
Distorted octahedral clusters formed by the hexanuclear cores in **1**.

The 5-nitropyridine-2-thiol anion works as a μ_3_-bridging ligand that links three different Cu(I) ions, one of which connected to the nitrogen and two to the axial μ_2_-sulfur. The progression of coppers bridged by the sulfur atoms results in a pair of staggered Cu_3_S_3_ rings, forming the distorted octahedral Cu_6_S_6_ cluster. The Cu···Cu distances in the Cu_6_S_6_ cluster are 2.792 and 2.819 Å (Figure 2), which are close to the sum of the van der Waals radii of two Cu(I) atoms (2.80 Å), thus indicating the presence of cuprophilic interactions within the cluster. X-ray powder diffractograms were collected form RT to 83 K, and no phase transition was observed (see Figure S2).

Taking the X-ray structure of **1** as initial guess, the geometry of the complex was optimized in vacuum, resulting in a highly symmetrical molecule belonging to the *D*_3d_ point group. It should be emphasized that the point-group symmetry found for **1** in the crystal is lower than the one calculated for an isolated cluster center in vacuum (*i.e.*, S_6_*vs D*_3d_, respectively). This can be easily attributed to the crystal anisotropy due to the hexagonal packing and because of the torsion of 10.45° between the nitro group and the pyridine ring of the organic ligand. Anyway, the non-coplanarity of the ligand does not significantly alter the ideal *D*_3d_ symmetry of **1**, as shown in Figure S3, where the theoretically computed structure and the experimental one are effectively overlapped with no substantial differences between the two geometries. As a result, the value of the minimized RMSD of all the atomic positions except hydrogen atoms is very low (*i.e.*, RMSD = 0.114 Å). For clarity, some key structural parameters of the two aforementioned geometries are highlighted and compared in Table S2.

### FT-IR Characterization

**Figure 3:**
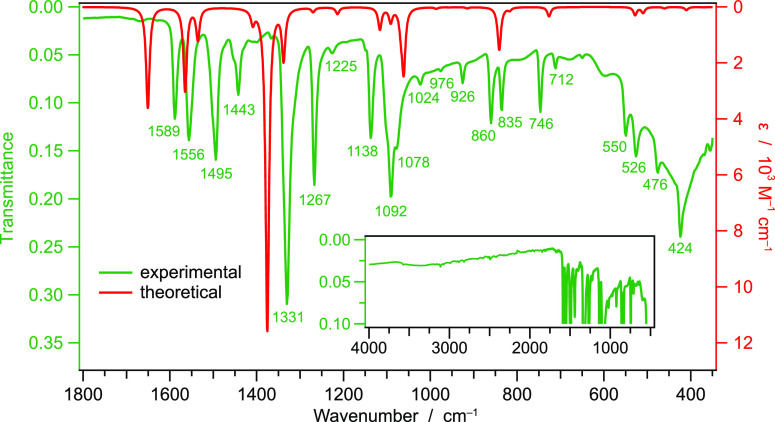
Comparison between the experimental (powder) and DFT-calculated infrared spectra of complex **1**. The theoretical spectrum is calculated in vacuum for a single cluster with *D*_3d_ point-group symmetry; the associated harmonic frequencies are empirically scaled by a factor of 0.95 (according to ref (47)), and the IR-active transitions are broadened using Lorentzian functions having a half width at half height of 5 cm^–1^. The inset reports the experimental IR spectrum up to 4000 cm^–1^, showing no signals attributable to solvated water molecules.

The FT-IR spectrum of compound **1** as neat powder shows characteristic peaks at 1589, 1495, 1443, and 1267 cm^–1^, which are attributed to stretching vibrations of the aromatic amine. The characteristic N–O asymmetric and symmetric stretching signals are at 1556 and 1331 cm^–1^, respectively. Bands at 1138 and 1092 cm^–1^ are due to C–H in-plane bending, while the band at 746 cm^–1^ is attributed to C–H out of plane bending. The interpretation of the FT-IR spectrum is in agreement with the harmonic vibrational frequencies computed in vacuum for **1** and reported in Figure 3. The lower energy bands at 550 and 526 cm^–1^ can be attributed to distortions in the Cu_6_S_6_ core, associated with C–S stretching and rigid vibrations of the nitropyridine moieties around the copper(I) core. The transitions at 476 and 424 cm^–1^ are almost exclusively assigned to out-of-plane vibrations within the pyridine rings. DFT vibrational analysis predicts normal modes primarily involving the Cu_6_S_6_ core only below 400 cm^–1^ and, therefore, such transitions are not visible in the experimental IR spectrum.

### Ground-State DFT Characterization

**Figure 4:**
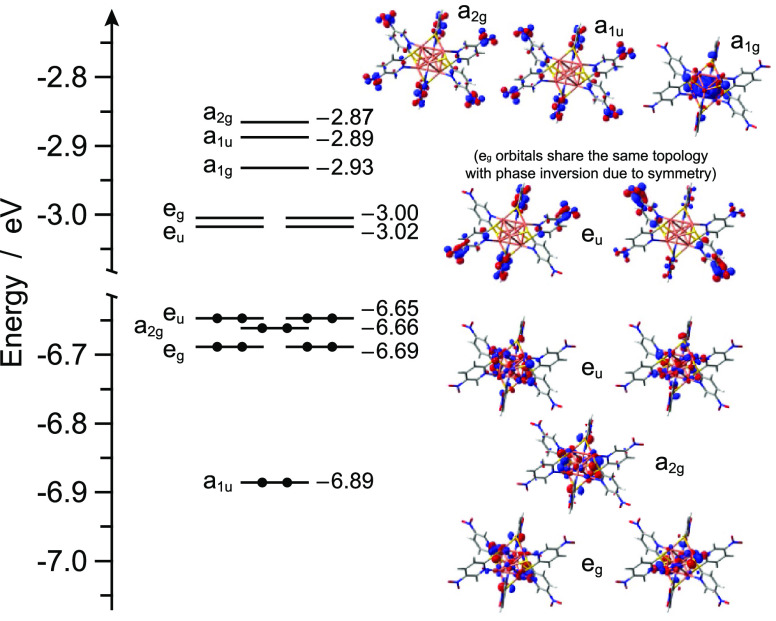
Energy diagram showing the frontier Kohn–Sham molecular orbitals of **1**. For some relevant orbitals, the corresponding isosurface is also displayed for the sake of clarity (isovalue = 0.04 × e^1/2^ bohr^–3/2^).

Figure 4 reports the energy diagram, together with the frontier molecular orbitals of **1**. The highest six occupied molecular orbitals are all cluster-centered mainly involving the copper(I) 3d orbitals and the 2p orbitals of the sulfur atoms. On the contrary, the lowest four unoccupied molecular orbitals are mainly localized on the π* nitro orbitals of the six equivalent organic ligands and are grouped in two couples of degenerate *e*_u_ and *e*_g_ orbitals; the remaining two orbitals involving the six π* nitro systems are the LUMO+5 and LUMO+6, having *a*_1u_ and *a*_2g_ symmetry. The LUMO+4 has a radically different nature, being a fully symmetric *a*_1g_ orbital with its maximum density right in the middle of the copper(I) distorted octahedron. Upper lying orbitals are much higher in energy (*i.e.*, 0.6 eV above LUMO+6) and, therefore, not further discussed.

### Photophysical characterization and DFT characterization of the excited states.

Absorption and emission properties of complex **1** have been determined in the solid state since the cluster structure was found to be unstable in solution.

A broad absorption tailing up to 700 nm has been observed in powder samples of **1** obtained from bulk solution (Figure S4), accounting for the deep red color of the solid material. The observed absorption features can be ascribed to low-energy excited states involving both cluster-centered and charge-transfer transitions.^48,49^

In order to get a deeper understanding of the complex excited-state scenario of compound **1**, singlet and triplet vertical excitations were computed by TD-DFT methods in vacuum (Tables S3 and S4). The lowest-energy transitions are attributed to cluster-centered excitations involving the copper(I) and sulfur atoms; they are grouped in two manifolds depending on their triplet or singlet multiplicity, which are located at (2.33 ± 0.06) eV for T_1_–T_5_ and at (2.5 ± 0.1) eV for S_1_–S_6_. Such transitions involve the excitation of one electron from the occupied orbitals, as reported in Figure 4, to the *a*_1g_ LUMO+4. On the other hand, at higher energies, two sets of charge-transfer transitions are encountered, involving an electron transfer from the cluster core (mainly from the sulfur 3p orbitals) to the π* nitropyridine orbitals: the triplet manifold is found at (2.60 ± 0.01) eV, with T_6_–T_11_ excited states, while 12 singlet charge-transfer excitations are computed at (2.91 ± 0.05) eV above S_0_ (Tables S3 and S4).

**Figure 5:**
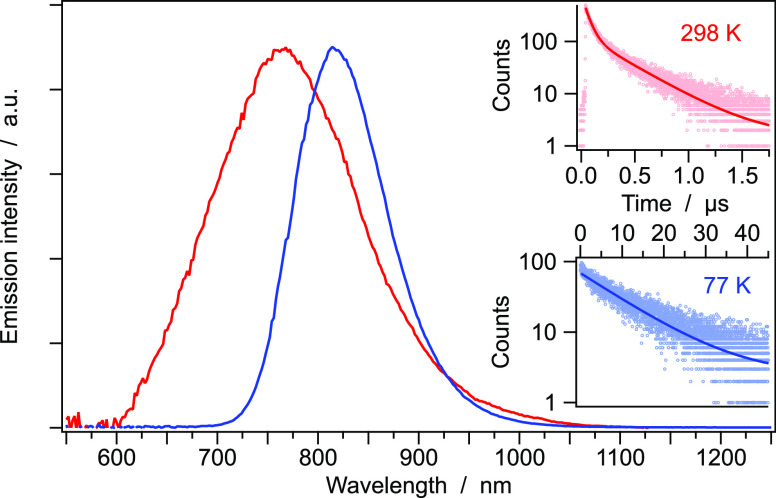
Normalized corrected emission spectra of complex **1** at room temperature (red) and at 77 K (blue), together with the associated excited-state lifetimes. Excitation wavelength: 407 nm.

Interestingly, complex **1** shows a remarkable unstructured red emission at room temperature with maximum at 765 nm (Figure 5), decaying to the ground state with a bi-exponential lifetime of 55 ns (28%) and 360 ns (72%). The estimated photoluminescence quantum yield is around 1%.^a^ It is worth noting that the emission spectrum collected at 298 K for complex **1** as a powder obtained in fast precipitation conditions (**1n**, see below for the discussion on the preparation conditions) was found to be bathochromically shifted by 40 nm with respect to the one prepared under solvothermal conditions (Figure S5). This result can be ascribed to the smaller dimension of the crystals and crystal domains in **1n**, as observed by the SEM images and the XRPD patterns (reported hereafter). The red-shift of the emission, in fact, can be attributed to loss of quantum confinement or photon propagation effects (self-absorption and re-emission)^50,51^ in the nanometric-sized crystals.

At 77 K, the emission spectrum sharpens and further red-shifts, showing its maximum at 815 nm (Figure 5). Notably, the excited-state lifetime becomes purely monoexponential and much longer if compared to the room-temperature one (*i.e.*, 11.1 *vs* 0.36 μs, respectively). Excitation spectra collected at both temperatures well overlay with the absorption profile, confirming the genuineness of the detected luminescence signals (Figure S4).

**Figure 6:**
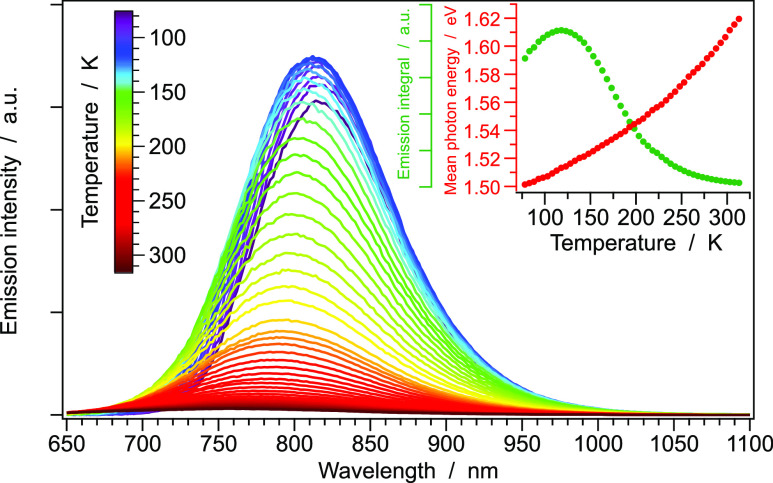
Emission spectra of **1** (as neat powder) recorded between 78 and 318 K. While a gradual increase in the mean-photon energy is observed upon increasing the temperature, a strong decrease in the emission intensity is also detected at *T* > 120 K.

In order to get a deeper understanding of the complex excited-state interplay occurring at different temperatures, the emission spectra and lifetimes of **1** (as neat powder) were recorded in the temperature range between 78 and 318 K. As reported in Figure 6, both the emission maximum and the output intensity are strongly temperature-dependent: when passing from 78 to 298 K, a blue-shift of the emission band of approximately 0.11 eV and a 20-fold decrease in intensity is observed.

It should be emphasized that the presence of only one broad emission band all across the investigated temperature range is rather uncommon for a Cu_6_S_6_ cluster equipped with N^S chelators. In fact, such clusters typically display two distinct emission bands at different temperatures: a high-energy band from charge-transfer cluster-to-ligand states and a lower-energy one with cluster-centered nature. For instance, a Cu_6_S_6_ analogue equipped with 6-methylpyridine-2-thiolate ligands displays a cluster-centered emission band at room temperature (λ_max_ = 690 nm), while high-energy charge-transfer bands become dominant at 5 K (λ_max_ = 480 nm).^48^ Moreover, when π-extended 1-methyl-1*H*-benzo[*d*]imidazole-2-thiolate ligands chelate the pseudo-octahedral cluster core, a considerable red-shift of the emission bands is reported.^49^ At room temperature, the corresponding Cu_6_S_6_ cluster displays a broad and unstructured emission centered at 875 nm, with a 1.7 μs lifetime and 0.01 quantum yield. Notably, upon cooling to 78 K, the emission splits into the higher-energy band at 750 nm and a lower-energy one at 970 nm; such two contributions were again attributed to cluster-to-ligand and cluster-centered states, respectively.^49^

On the contrary, the totally different scenario observed for **1** resembles the one typical of other copper(I) complexes, displaying thermally activated delayed fluorescence, like dinuclear Cu_2_I_2_ compounds in which an interplay between T_1_ and S_1_ is observed.^8^ Anyway, the present case is much more complicated due to the high symmetry of the Cu_6_S_6_ cluster; accordingly, the presence of five lowest-lying cluster-centered triplet states must be considered, together with the corresponding nearby singlet manifold (Tables S3 and S4, respectively). On the other hand, the involvement of higher-energy charge-transfer states is really improbable since it would have led to the above-discussed double emission bands (which are generally separated by 0.4 eV), while in our case, only a minor band shift of about 0.1 eV is observed (Figures 5 and 6).

**Figure 7:**
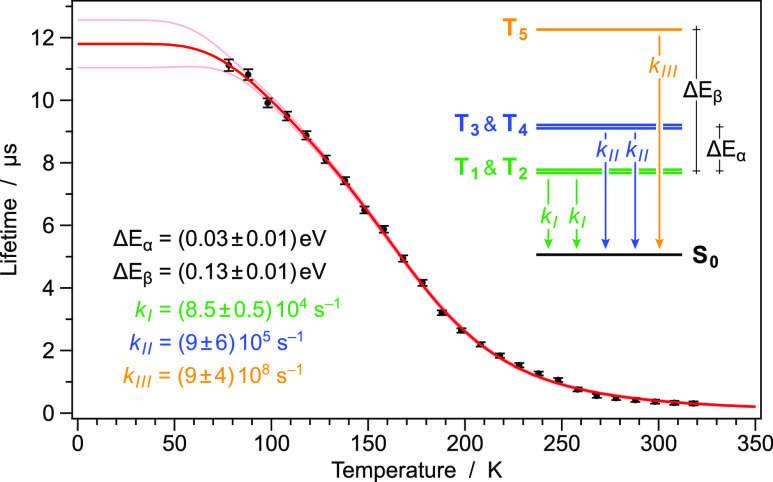
Temperature-dependent excited-state lifetimes of **1** (as neat powder) recorded between 78 and 318 K (black dots). The red curve represents the fit according to eq 2, using the reported optimized parameters (bottom left), according to the proposed energy-level scheme (top right). Parameters are given with 90% confidence interval; reduced χ^2^ = 1.27.

Figure 7 reports the temperature-dependent analysis of the excited-state lifetime of **1**. When temperature is increased, the emission decay time is drastically reduced (Figure 7), with a corresponding drop in the quantum yield (Figure 6). Accordingly, the estimated radiative constant at 298 K remains comparable to that at 78 K (*i.e.*, *k*_r_ ≈ 3 *vs* 2 × 10^4^ s^–1^), suggesting an interplay of states with a similar nature (*i.e.*, cluster-centered triplets). On the contrary, the non-radiative constant (*k*_nr_) is estimated to be 6 × 10^4^ s^–1^ at 78 K, and it increases 50 times at 298 K [*i.e.*, *k*_nr_ (298 K) ≈ 3 × 10^6^ s^–1^]. Such a trend in the *k*_r_ and *k*_nr_ values suggests that the lifetime shortening upon temperature increase is not due to the population of upper-lying singlet excited states (which would have led to an increase in the *k*_r_ values at higher temperatures) but is more likely due to the thermal activation of vibrational modes that couple with S_0_, which are particularly important non-radiative pathways in the NIR region.

The temperature dependence of the emission decay time allows the evaluation of an energy-level diagram for the spectroscopically active states involved in the emission of **1**. Assuming a fast thermalization, the occupation dynamics of excited states is governed by the formula:
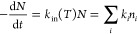
1where *N* is the total number of occupied excited states and *k*_in_ is the total rate constant for depopulation of the equilibrated system of excited states at a defined temperature (*i.e.*, the inverse of the measured lifetimes, as reported in Figure 7). Specifically, *n*_*i*_ is the Boltzmann occupation, and *k*_*i*_ is the total rate constant for depopulation of the particular state *i*.^52,53^ Introduction of Boltzmann factors, within the framework of above-made considerations, leads to an expression that can be applied to fit the measured lifetime data, as reported in Figure 7
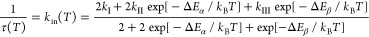
2where *k*_B_ is the Boltzmann constant, and Δ*E*_α_ and Δ*E*_β_ are the energy differences between the two degenerate *E*_g_ triplets (T_3_ and T_4_) and the lowest-energy *E*_u_ couple (T_1_ and T_2_), and between the upper-lying *A*_2g_ triplet T_5_ and the *E*_u_ couple, respectively. The energy order and symmetry attribution have been accomplished according to TD-DFT calculations (see above and Table S4), without considering the excited-state relaxation and symmetry lowering of such cluster-centered triplet states. It should be emphasized that the energy splitting between T_5_ and the lowest-energy *E*_u_ couple calculated from the temperature-dependent lifetime fitting procedure (Δ*E*_β_) is in good agreement with the spectral shift occurring on heating from 78 to 298 K (*i.e.*, 0.13 *vs* 0.11 eV). Furthermore, the fitting procedure gives values for the intrinsic decay times of all the five cluster-centered triplets, as reported in Figure 7.

It is remarked that it cannot be definitely excluded that more than these five triplet states are involved in the emission process since TD-DFT calculations estimate four of the lowest six singlet states of cluster-centered nature to be nearly isoenergetic with T_5_ at the Franck–Condon region (compare Tables S3 and S4). Therefore, eq 2 should be considered as the simplest model capable of fitting the experimental data, in accordance with DFT predictions.

**Figure 8:**
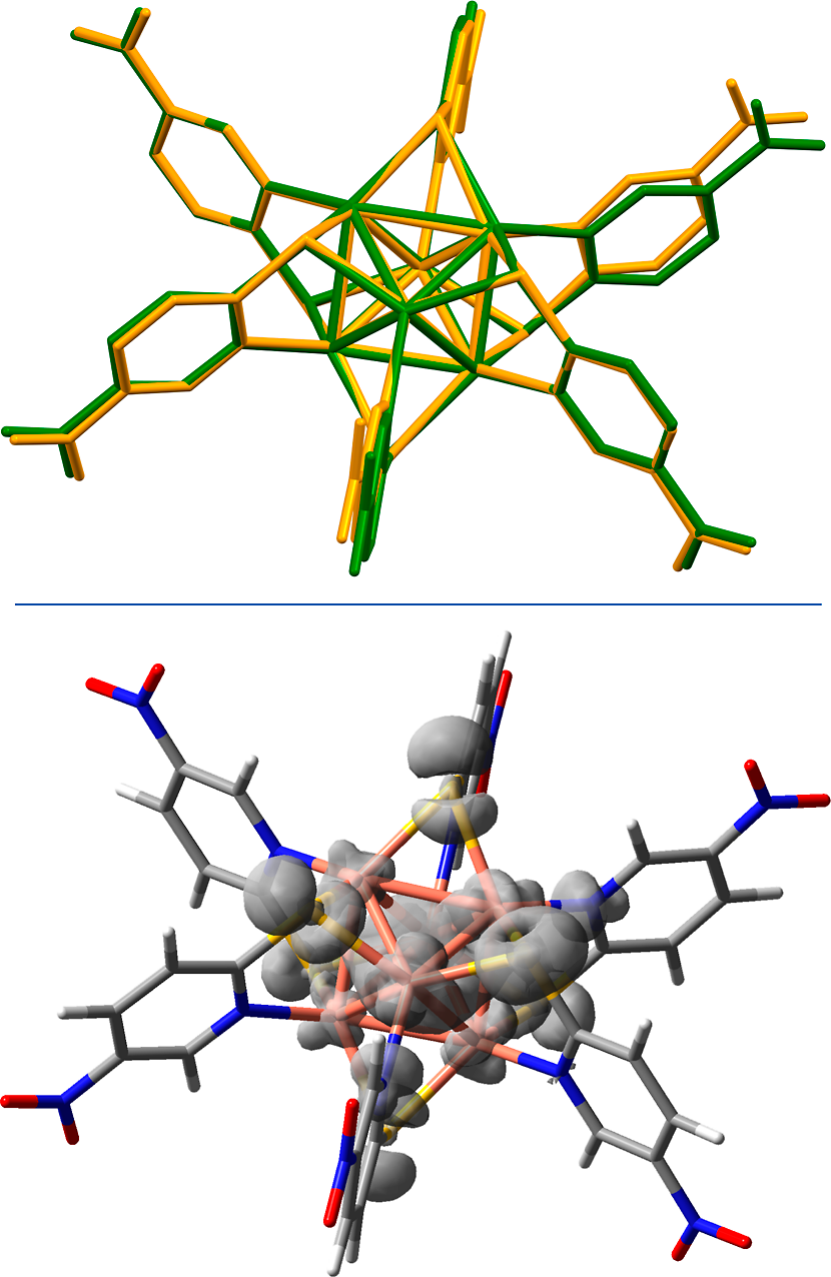
(Top) Comparison between the ground-state minimum-energy geometry of complex **1** (green) and the one of the lowest triplet state T_1_ (orange). The structural overlap is obtained by minimizing the RMSD of the Cu_6_S_6_-cluster atoms. (Bottom) The spin-density distribution of T_1_ is also reported, showing the cluster-centered nature of such excited states.

Unrestricted DFT optimizations (carried out starting from the ground-state geometry and imposing a spin multiplicity of 3) have also been carried out to better elucidate the nature of the lowest triplet state, which is mainly responsible for the emission of complex **1**. According to the irreducible representations of the lowest five triplet excited states (Table S4), a symmetry lowering is always expected to occur upon triplet relaxation. Indeed, as shown in Figure 8, the highly symmetrical minimum-energy geometry of *S*_0_ (displaying *D*_3d_ symmetry) is compared to the one of T_1_, which was found to belong to the *C*_s_ point group; the key structural parameters are also summarized in Table S2.

As demonstrated by the spin-density distribution of T_1_ (Figure 8, bottom), this excited state displays a cluster-centered nature. In its relaxed triplet state, the hexanuclear copper core experiences a considerable contraction, with an average Cu–Cu bond length of (2.57 ± 0.06) Å, to be compared to a calculated ground-state length of 2.75 Å (Figure 8, top). Such a cage shrink is due to the population of the LUMO+4 (see Table S4), which is a totally symmetric *a*_1g_ orbital made up by the fully bonding combination of the six copper(I) 4s/4p hybrids (see Figure 4).

### Use of Different Preparation Conditions

**Figure 9:**
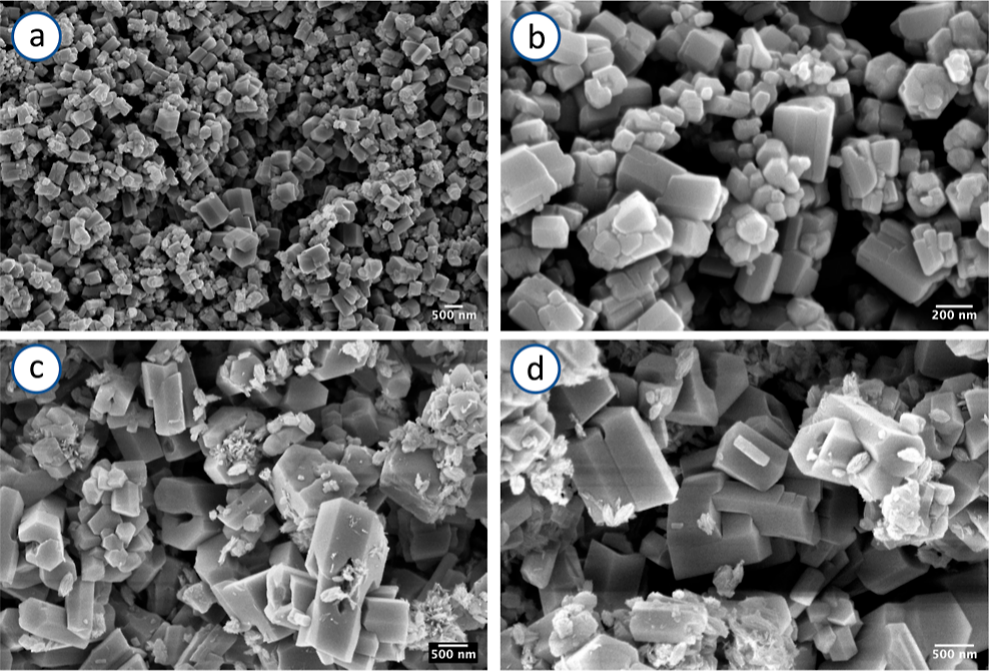
SE SEM images of **1n** (a,b) and bulk **1** (c,d), at different magnifications, beam energy *E* = 5 keV.

Interestingly, modification of the reaction conditions, that is, using a fast precipitation method (see the Experimental Methods section for details) gives rise to the formation of crystals of **1** with sizes in the nanometric range (**1n**). XRPD analyses confirm that the structure of **1n** corresponds to that of **1** (Figure S1). The significant differences in the micrometric–submicrometric structures of **1n** and bulk **1** have been identified by SEM analysis (Figure 9). SEM images of **1n** (Figure 9a,b) show an inhomogeneous distribution of nanoprisms with lengths and widths in a range of 60–200 nm, whereas bulk **1** (Figure 9c,d) consists of larger particles with dimensions of 0.6–2 μm. Pawley refinement allowed us to determine the average crystal domain by the Scherrer formula based on the integral breadth,^35^ which resulted in 110 and 55 nm for **1** and **1n**, respectively (Figures S6 and S7), in line with SEM observations.

## Conclusions

A novel Cu_6_S_6_ cluster with a peculiar emission in the NIR region has been synthesized and characterized. In the cluster, each copper atom is connected to two sulfur atoms and one nitrogen atom from three different 5-nitropyridine-2-thiol molecules, the latter working as μ_3_-bridging ligands between three different Cu(I) ions. The resulting distorted octahedral Cu_6_S_6_ cluster crystallizes in the trigonal *R*3® space group. The cluster shows a temperature-dependent luminescence, with a broad emission band peaking at 765 nm at room temperature which sharpens, increases in intensity, and red-shifts up to 815 nm at 77 K. The variation in the spectral features and lifetime with temperature has been analyzed and interpreted by means of TD-DFT calculations, revealing a complex interplay of at least five triplet states involved in the emission process. Interestingly, the preparation conditions, that determines the micrometric–submicrometric structure of the compound, have been found to affect the emission features, opening new perspectives for the development of NIR emissive Cu(I) materials.

## Supporting Information Available

Additional crystallographic and spectroscopic data (PDF)

## Footnotes

a The absolute emission quantum yield of **1** was found to be below the limit of detection of the system, that is, 2%.
