# Supplementary material for: Novel Cu(I)-5-nitropyridine-2-thiol Cluster with NIR Emission: Structural and Photophysical Characterization
Source: J Phys Chem C Nanomater Interfaces. 2022 Jun 8;126(24):10190–8. doi: 10.1021/acs.jpcc.2c01842 (PMC9234981; doi:10.1021/acs.jpcc.2c01842)
Supplement: Supplementary file 1 — jp2c01842_si_001.pdf [file jp2c01842_si_001.pdf]

Supporting Information for

A Novel Cu(I)-5-nitropyridine-2-thiol Cluster with NIR Emission: Structural and Photophysical Characterization

Khaled Hassanein,[a] Chiara Cappuccino,[b] Marianna Marchini,[b] Elisa Bandini,[a] Meganne Christian,[c] Vittorio Morandi,[c] Filippo Monti,[a] Lucia Maini,[b*] Barbara Ventura[a*]*

[a] Istituto per la Sintesi Organica e la Fotoreattività (ISOF), Consiglio Nazionale delle Ricerche (CNR), Via P. Gobetti 101, 40129 Bologna, Italy. email: filippo.monti@isof.cnr.it; barbara.ventura@isof.cnr.it

[b] Dipartimento di Chimica "G. Ciamician", Università di Bologna, Via F. Selmi 2, 40126 Bologna, Italy. email: l.maini@unibo.it

[c] Istituto per la Microelettronica e Microsistemi (IMM) Sede di Bologna, Consiglio Nazionale delle Ricerche (CNR), Via P. Gobetti 101, 40129 Bologna, Italy.

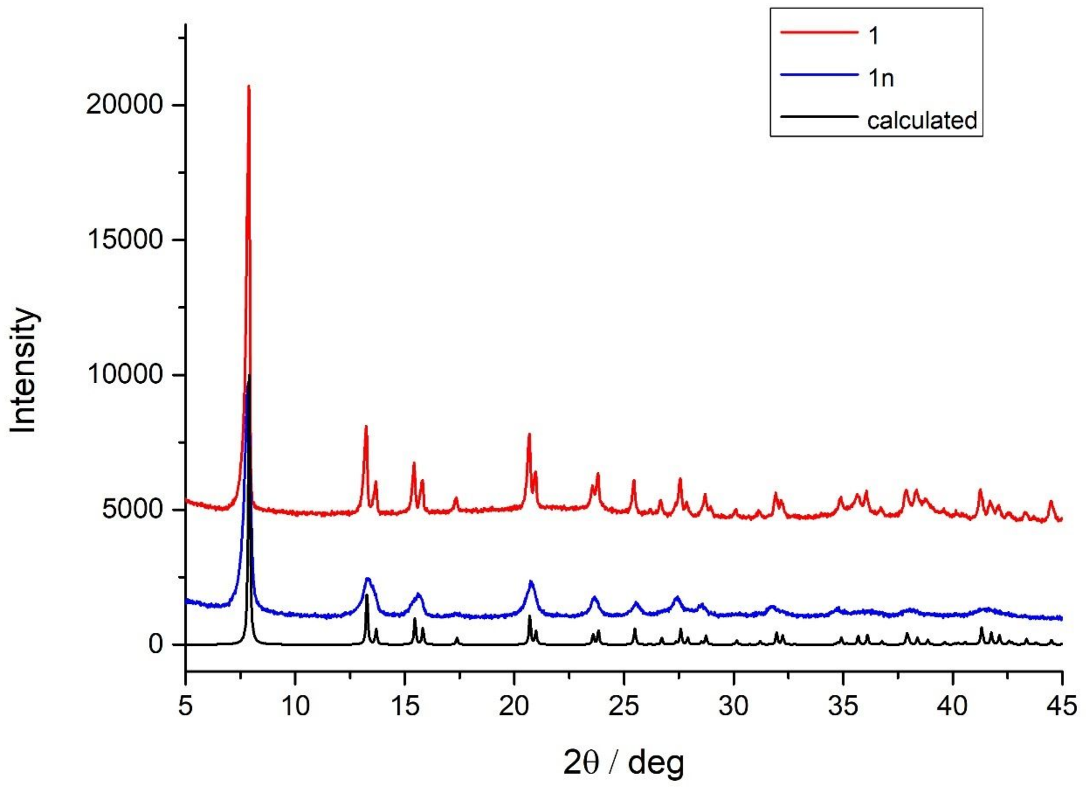

Figure S1: XRPD patterns of **1**, **1n** and the calculated pattern of **1.**

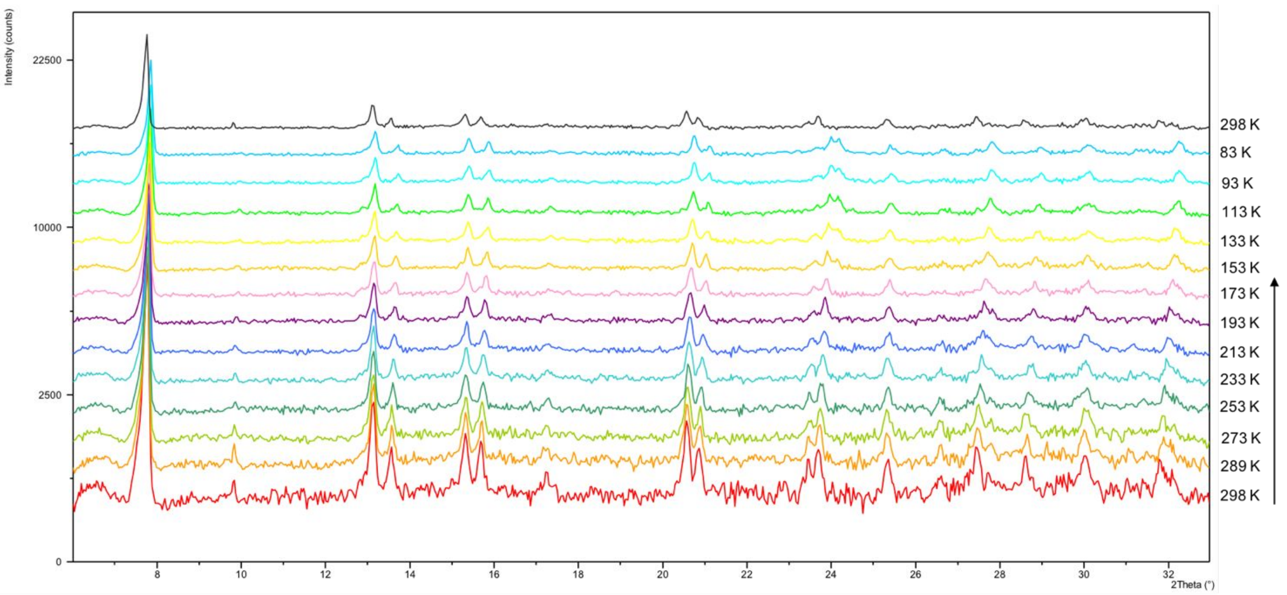

Figure S2. Variable-temperature X-ray powder diffraction analysis of compound **1**. Powder pattern profiles measured *in situ*, color changes of the sample cooled down from 298 K (red line, bottom) to 83 K. At the end of the measurement, the sample was heated up to 298 K again (black line, top). No phase transition is observed, the shift of the peaks is due to thermal contraction.

Table S1. Crystallographic data of compound **1** at room temperature.

Chemical formula	$C_{30} H_{18} Cu_6 N_{12} O_{12} S_6$
Formula Mass	1312.22 g/mol
Temperature	RT
Crystal System	Trigonal
Space Group	R-3
a/Å	22.3832(12)
b/Å	22.3832(12)
c/Å	7.0996(4)
α/°	90
β/°	90
γ/°	120
Volume/ $Å^3$	3080.41
R(int)	0.035
R1	0.0342
R2 (all data)	0.0530
Chi^2	1.013

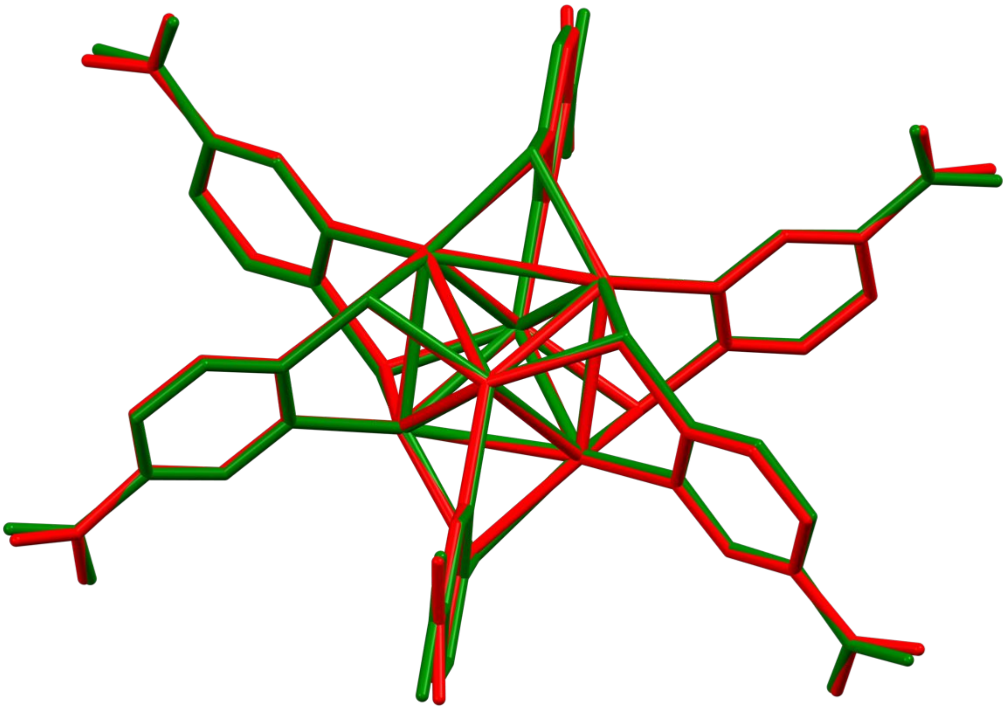

Figure S3. Comparison between the experimental X-ray structure of complex **1** (red) and the one optimised by DFT methods (green). The structural overlap is obtained by minimizing the root-mean-square deviation (RMSD) of all the atomic positions except hydrogen atoms.

Table S2. Comparison between key geometrical parameters of complex **1** obtained from X-ray diffraction crystallography and DFT optimizations in vacuum.

	X-ray data [S_6]	DFT data ground state [D_{3d}]	U-DFT data lowest triplet state [C_s]
Distances [Å]			
Cu–Cu	2.819	2.757	2.582–2.438–2.582<br>2.596–2.568–2.596
Cu–Cu’	2.792	2.751	2.635–2.620–2.563
Cu–S	2.249–2.250	2.284	2.340–2.363–2.371<br>2.330–2.341–2.338
Cu–N	2.053	2.079	2.009–2.069–2.009<br>2.099–2.102–2.099
C–S	1.736	1.750	1.751–1.751–1.751<br>1.746–1.747–1.746
Angles [°]			
Cu–Cu–Cu	60.00	60.00	61.83–56.34–61.83<br>60.35–59.29–60.35
Cu–Cu’–Cu	60.66	60.12	58.86–56.80–58.86<br>60.10–58.33–60.10
Cu–Cu–Cu’	59.67	59.94	60.86–61.60–60.28<br>60.84–61.03–58.87
Cu–S–Cu	77.62	74.22	66.59–61.88–66.59<br>67.38–66.87–67.38
S–Cu–Cu	51.19–51.21	52.89	56.28–59.06–57.13<br>56.26–56.56–56.36
S–Cu–Cu’	79.99–80.00	82.53	86.91–87.31–86.79<br>84.69–85.77–83.37
N–Cu–Cu	142.00–143.38	142.25	143.87–141.25–148.17<br>143.98–140.77–142.82
N–Cu–Cu’	86.50–87.34	86.06	87.00–88.28–91.25<br>85.37–86.28–87.84
Cu–N–C	120.68	122.38	121.25–123.14–121.25<br>124.23–123.50–124.23
Cu–S–C	105.94–106.49	104.27	101.35–100.46–101.09<br>102.45–100.80–101.75
N–Cu–S	110.22–111.78	110.91	110.27–102.64–106.69<br>109.63–106.18–106.55
S–C–N	119.61	119.70	120.45–120.51–120.45<br>120.92–120.67–120.92
Dihedrals [°]			
Cu–N–C–S	1.30	0.00	2.08–0.00–2.08<br>2.40–0.00–2.40
Cu–Cu’–S–C	44.27–44.94	43.40	40.50–40.95–38.71<br>40.36–42.36–38.14
Cu–Cu–Cu–S	25.30	27.58	31.15–32.28–31.56

			27.83–30.09–27.95
Cu–Cu–Cu–S	148.00	146.02	143.52–144.74–143.52<br>147.96–145.36–147.96
Cu’–Cu’–Cu’–N	138.46–140.02	138.22	137.18–144.57–150.76<br>136.43–138.80–141.52
Cu–Cu–Cu–Cu’	54.33	54.66	55.09–55.54–55.79<br>54.48–55.59–56.28
Cu–Cu–Cu–Cu’	109.74	109.52	109.32–110.07–109.32<br>108.09–109.14–108.09
py–NO_2	10.45	0.00	0.23–0.00–0.23<br>0.04–0.00–0.04

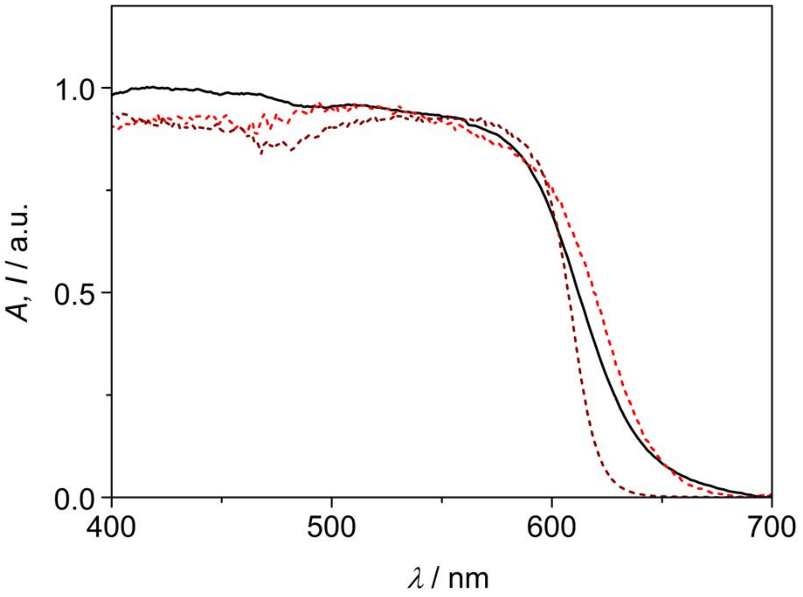

Figure S4. Arbitrarily scaled absorption spectrum (black solid), room temperature excitation spectrum (red dash, λ_{em} = 768 nm), low temperature excitation spectrum (brown dash, λ_{em} = 823 nm) of a powder sample of **1** obtained from bulk solution.

Table S3. The lowest 18 singlet vertical excitation for compound **1** in vacuum. Density differences are computed as $\rho_{Sn} - \rho_{S0}$ using relaxed excited-state densities and the ground-state SCF one. Isodensities = + 0.0004 (yellow, electron) and – 0.0004 e bohr^{-3} (gray, hole). The symmetry of each excited state (and molecular orbital) is given accordingly to the ground-state D_{3d} point group.

	Transition energy [eV (nm)] *oscillator strength*	Density difference ($\rho_{Sn} - \rho_{S0}$)	Molecular-orbital contribution	Nature
**$S_0 \rightarrow S_1$** (E_u)	2.42 (512) *f = 0.092*	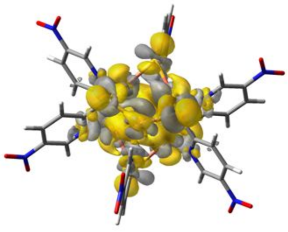	H–1 → L+4 (e_u) (a_{1g}) 96.1%	cluster centered
**$S_0 \rightarrow S_2$** (E_u)	2.42 (512) *f = 0.092*	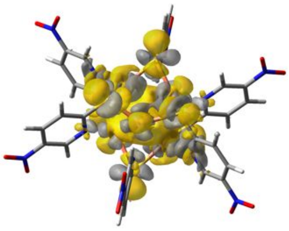	H → L+4 (e_u) (a_{1g}) 96.1%	cluster centered
$S_0 \rightarrow S_3$ (E_g)	2.43 (511) *f = 0.000*	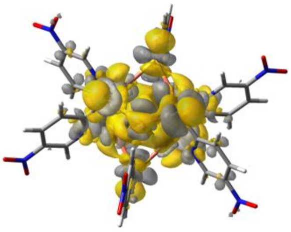	H–4 → L+4 (e_g) (a_{1g}) 97.6%	cluster centered
$S_0 \rightarrow S_4$ (E_g)	2.43 (511) *f = 0.000*	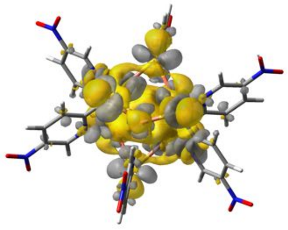	H–3 → L+4 (e_g) (a_{1g}) 97.6%	cluster centered
$S_0 \rightarrow S_5$ (A_{2g})	2.49 (499) *f = 0.000*	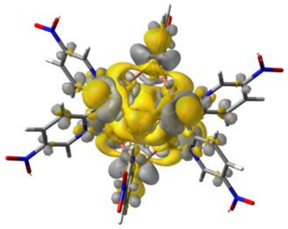	H–2 → L+4 (a_{2g}) (a_{1g}) 97.5%	cluster centered
$S_0 \rightarrow S_6$ (A_{1u})	2.80 (443) *f = 0.000*	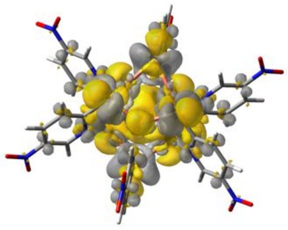	H–5 → L+4 (a_{1u}) (a_{1g}) 93.1%	cluster centered

$S_0 \rightarrow S_7$ (E_u)	2.83 (438) *f* = 0.000	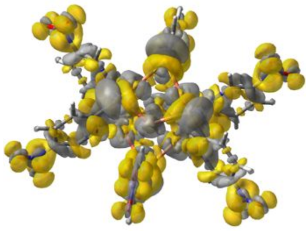	highly multiconfigurational character	charge transfer from core (mainly S) to nitropyridines
$S_0 \rightarrow S_8$ (E_g)	2.83 (438) *f* = 0.000	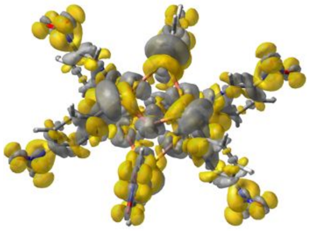	highly multiconfigurational character	charge transfer from core (mainly S) to nitropyridines
$S_0 \rightarrow S_9$ (E_u)	2.88 (431) *f* = 0.000	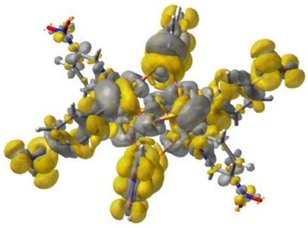	highly multiconfigurational character	charge transfer from core (mainly S) to nitropyridines
$S_0 \rightarrow S_{10}$ (E_u)	2.88 (431) *f* = 0.000	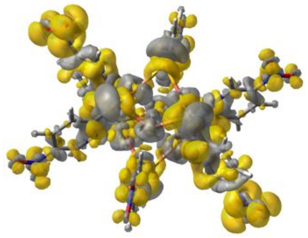	highly multiconfigurational character	charge transfer from core (mainly S) to nitropyridines
$\mathbf{S_0 \rightarrow S_{11}}$ (E_g)	2.89 (430) *f* = 0.001	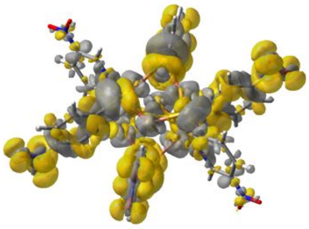	highly multiconfigurational character	charge transfer from core (mainly S) to nitropyridines
$\mathbf{S_0 \rightarrow S_{12}}$ (E_g)	2.89 (430) *f* = 0.001	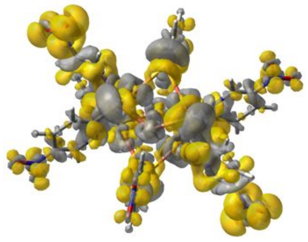	highly multiconfigurational character	charge transfer from core (mainly S) to nitropyridines
$S_0 \rightarrow S_{13}$ (E_u)	2.93 (423) *f* = 0.000	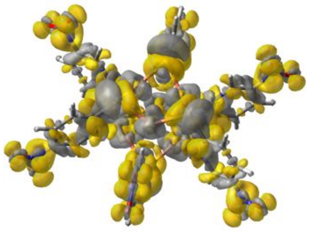	highly multiconfigurational character	charge transfer from core (mainly S) to nitropyridines

$S_0 \rightarrow S_{14}$ (E_g)	2.94 (422) *f* = 0.000	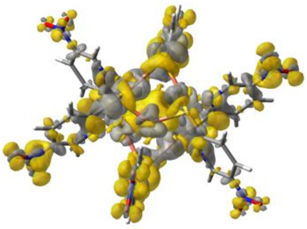	highly multiconfigurational character	mixed cluster centered and charge transfer nature
$S_0 \rightarrow S_{15}$ (E_g)	2.94 (422) *f* = 0.000	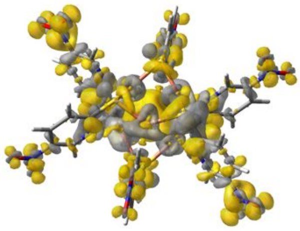	highly multiconfigurational character	mixed cluster centered and charge transfer nature
$\mathbf{S_0 \rightarrow S_{16}}$ (E_g)	2.94 (422) *f* = 0.015	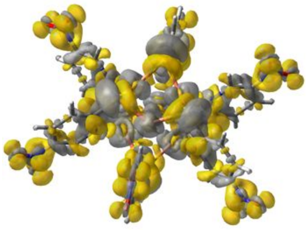	highly multiconfigurational character	charge transfer from core (mainly S) to nitropyridines
$\mathbf{S_0 \rightarrow S_{17}}$ (E_g)	2.96 (419) *f* = 0.094	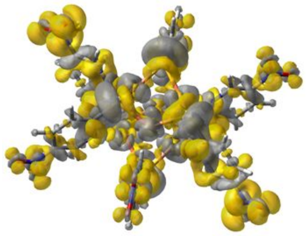	highly multiconfigurational character	charge transfer from core (mainly S) to nitropyridines
$\mathbf{S_0 \rightarrow S_{18}}$ (E_g)	2.96 (419) *f* = 0.094	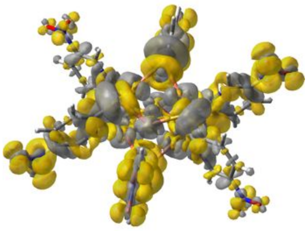	highly multiconfigurational character	charge transfer from core (mainly S) to nitropyridines

Table S4. The lowest 14 triplet vertical excitation for compound **1** in vacuum. Density differences are computed as $\rho_{Tn} - \rho_{S0}$ using relaxed excited-state densities and the ground-state SCF one. Isodensities = + 0.0004 (yellow, electron) and – 0.0004 e bohr^{-3} (gray, hole). The symmetry of each excited state (and molecular orbital) is given accordingly to the ground-state D_{3d} point group.

	Transition energy [eV (nm)]	Density difference ($\rho_{Tn} - \rho_{S0}$)	Molecular-orbital contribution	Nature
$S_0 \rightarrow T_1$ (E_u)	2.27 (547)	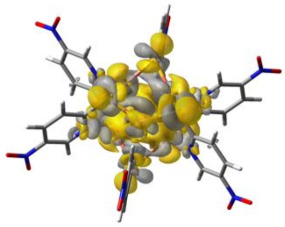	H–1 (e_u) → L+4 (a_{1g}) 96.8%	cluster centered

$S_0 \rightarrow T_2$ (E_u)	2.27 (547)	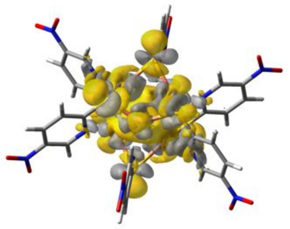	H (e_u) → L+4 (a_{1g}) 96.8%	cluster centered
$S_0 \rightarrow T_3$ (E_g)	2.35 (528)	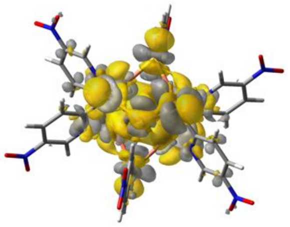	H–4 (e_g) → L+4 (a_{1g}) 97.1%	cluster centered
$S_0 \rightarrow T_4$ (E_g)	2.35 (528)	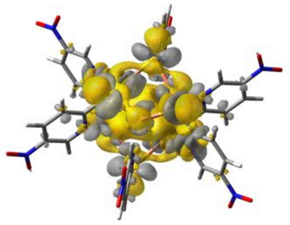	H–3 (e_g) → L+4 (a_{1g}) 97.1%	cluster centered
$S_0 \rightarrow T_5$ (A_{2g})	2.41 (514)	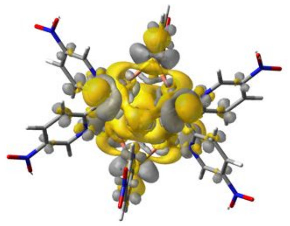	H–2 (a_{2g}) → L+4 (a_{1g}) 96.8%	cluster centered
$S_0 \rightarrow T_6$ (E_g)	2.60 (477)	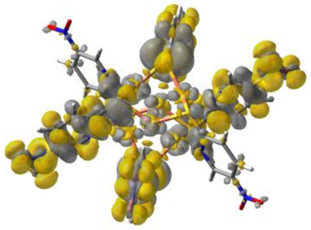	highly multiconfigurational character	charge transfer from core (mainly S) to nitropyridines
$S_0 \rightarrow T_7$ (E_g)	2.60 (477)	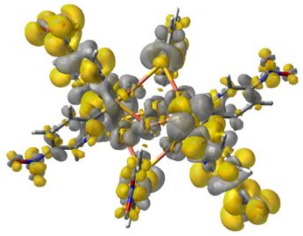	highly multiconfigurational character	charge transfer from core (mainly S) to nitropyridines
$S_0 \rightarrow T_8$ (E_u)	2.60 (477)	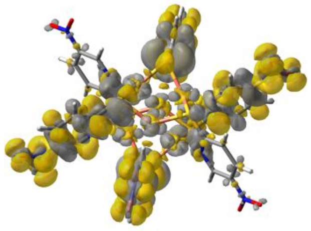	highly multiconfigurational character	charge transfer from core (mainly S) to nitropyridines

$S_0 \rightarrow T_9$ (E_u)	2.60 (477)	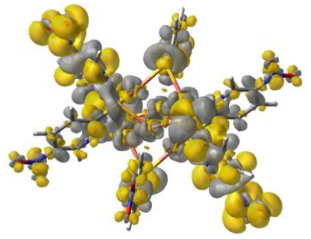	highly multiconfigurational character	charge transfer from core (mainly S) to nitropyridines
$S_0 \rightarrow T_{10}$ (E_g)	2.61 (476)	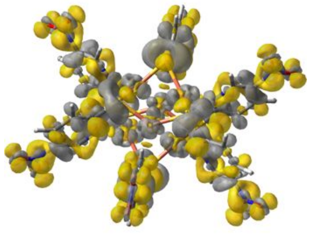	highly multiconfigurational character	charge transfer from core (mainly S) to nitropyridines
$S_0 \rightarrow T_{11}$ (E_u)	2.61 (474)	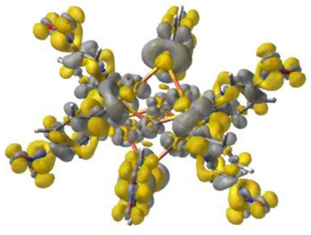	highly multiconfigurational character	charge transfer from core (mainly S) to nitropyridines

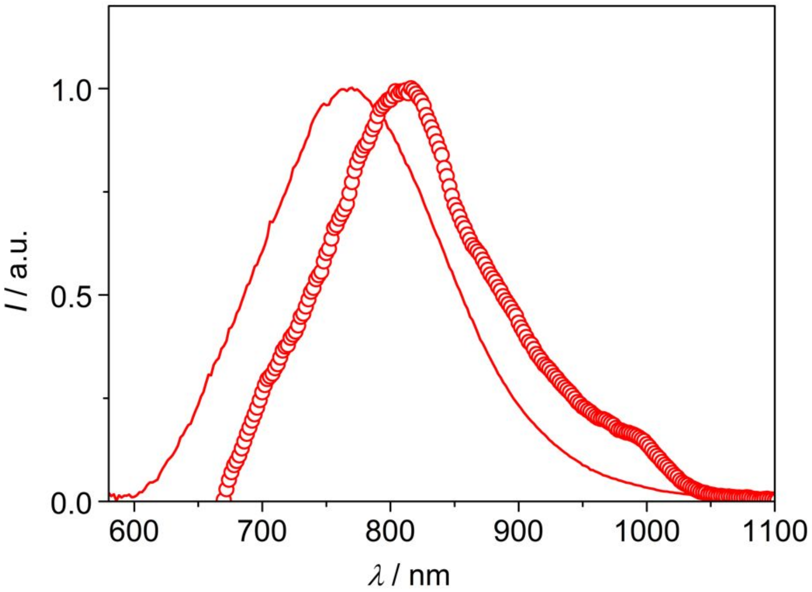

Figure S5. Normalized corrected emission spectra of **1** (as powder) from bulk solution (solid line) and from fast precipitation conditions (**1n**, open circles). λ_{exc} = 450 nm.

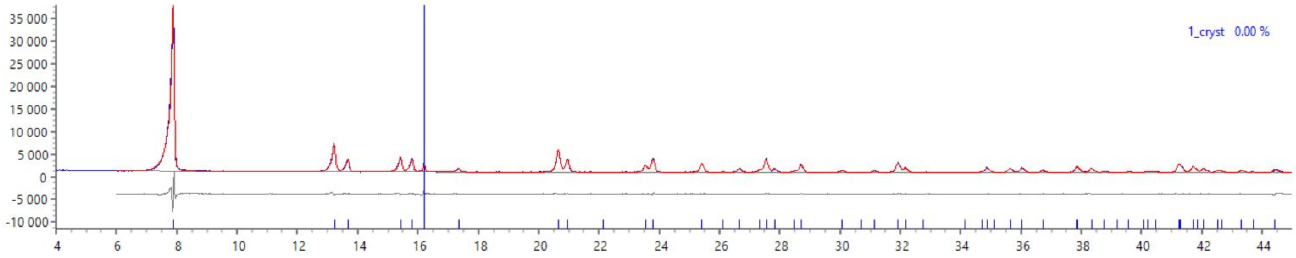

Figure S6. Pawely refinement on the powder patter of the sample **1** to determine the average crystal size with the Scherrer formula (crystal shape factor =1). An extra peak due the sample holder is observed at 2θ= 16.21°. Experimental pattern in blue, calculated pattern in red, difference plot in gray. The refinement converged at R_{wp}=4.53%.

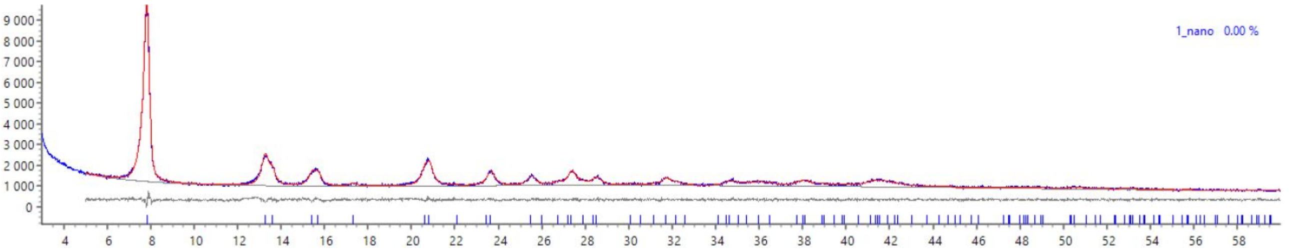

Figure S7. Pawely refinement on the powder patter of the sample **1n** to determine the average crystal size with the Scherrer formula (crystal shape factor =1). Experimental pattern in blue, calculated pattern in red, difference plot in gray. The refinement converged at R_{wp}=2.81%.
